# Herbal medicines for metabolic diseases with blood stasis: A protocol for a systematic review and meta-analysis

**DOI:** 10.1097/MD.0000000000014543

**Published:** 2019-02-22

**Authors:** Mi Mi Ko, Soobin Jang, Jeeyoun Jung

**Affiliations:** Clinical Medicine Division, Korea Institute of Oriental Medicine, Daejeon, Republic of Korea.

**Keywords:** blood stasis, herbal medicine, Metabolic disease, systematic review

## Abstract

**Background:**

Herbal medicines have the potential to be alternative treatments for metabolic diseases. This systematic review will assess the efficacy of herbal medicines in treating metabolic diseases with blood stasis.

**Methods and analysis:**

We will search MEDLINE, the Cochrane Central Register of Controlled Trials (CENTRAL), Web of Science, 2 Korean medical databases (OASIS, NDSL), a Chinese database (China National Knowledge Infrastructure, CNKI), and a Japanese database (J-STAGE) for relevant literature. We will include all randomized controlled trials (RCTs) or quasi-RCTs evaluating the effectiveness of herbal medicine. Participants of both sexes and of any age with clinically diagnosed metabolic disease with blood stasis will be included. Primary outcomes will include Blood-stasis syndrome score, TC, TG, HDL-C and LDL-C. Secondary outcomes will Blood pressure, FBS. ECG, prevalence rate of heart disease, and response rate. The risk of bias will be assessed using the Cochrane tool for assessing risk of bias.

**Discussion:**

The findings of this study will provide a summary of the current state of evidence regarding the effectiveness of types of herbal medicine in managing metabolic disease with blood stasis. In addition, this review will be expected to provide a base for clinical trials to confirm the efficacy of herbal medicine for treatments of metabolic disease with blood stasis.

**Trial registration number:**

PROSPERO 2018 CRD42018090179.

## 1 Introduction

Metabolic disease and metabolic syndrome refer to diseases that are associated with an abnormality of metabolism like glucose, fat, or protein and include cancer, diabetes, bone metabolic disease, fatty liver, obesity, and cardiovascular disease, etc.^[1–5]^ Metabolic syndrome is diagnosed when 3 or more out of 5 criteria are met.^[2–7]^ Insulin resistance is considered the fundamental cause of metabolic syndrome. If resistance increases, insulin secretion will be excessive, leading to metabolic syndrome. In most cases, insulin resistance is associated with obesity, stress, decreased physical activity, autonomic hyperactivity, and hereditary factors.^[3–5]^

The number of patients with metabolic disease is increasing in association with population aging, and the demand for drugs to treat metabolic diseases will increase accordingly.^[6,7]^ In Korea, the prevalence of metabolic di**s**eases increased from 16.1% in 2007 to 17.9% in 2013. The National Nutrient Health Survey showed that the prevalence of metabolic syndrome among adults aged 30 or more in Korea is 28.8%.^[8]^

Metabolic syndrome can be primarily alleviated by correcting lifestyles and habits, but in many cases, medication treatment is inevitable as some of the symptoms will remain.^[2–6]^ However, no drug has been approved for long-term treatment of metabolic syndrome.^[9]^ In addition, a new treatment strategy is needed to alleviate various side effects caused by polypharmacy required for the diverse symptoms of metabolic syndrome.^[9,10]^ Statins have monopolized the market for cardiovascular disease. However, some statins reportedly have digestive system side effects, and efforts are being made to develop alternative drugs to ensure patient safety.^[11,12]^ Moreover, as the interest in new drugs with fewer side effects and improved efficacy increases, it is expected that research and investment in the development of drugs based on natural substances will be expanded.^[5,13–15]^

In traditional Korean medicine, it has been reported that treating blood stasis is effective in treating obesity and metabolic diseases.^[16,17]^ Heat in blood, thick blood, and blood stasis or phlegm arising therefrom are considered the cause of metabolic syndrome. When blood is heated, it becomes sticky and thickens. Thick blood is circulated throughout the body, heating organs and inducing by products such as blood stasis or phlegm. Such by products eventually block the circulation of Qi-blood and increase internal body temperature, leading to the symptoms of metabolic syndrome.^[16–18]^ Some studies including in vivo and in vitro studies have examined treatments to alleviate the symptoms of metabolic disease through treatment of blood stasis.^[5,19–21]^ These treatments are based on the principles of traditional Korean medicine, but clinical research is needed to verify the utility of prescriptions for treatment of blood stasis. Some review papers have examined herbal medicine for treatment of metabolic diseases, but no systematic literature review has focused on the cardiovascular system.^[5]^

Thus, the present systematic review hereby aims to evaluate the efficacy of herbal medicine for treatment of metabolic diseases, with a focus on the cardiovascular system.

## 2 Methods

### 2.1 Study registration

This protocol review has been registered on PROSPERO 2018 CRD42018090179 (Available from: http://www.crd.york.ac.uk/PROSPERO/display_record.php?ID=CRD42018090179).

### 2.2 Data source

We will search MEDLINE, the Cochrane Central Register of Controlled Trials (CENTRAL), Web of Science, 2 Korean medical databases (OASIS, NDSL), a Chinese database (China National Knowledge Infrastructure, CNKI), and a Japanese database (J-STAGE) for relevant literature. We will use the following search terms: (cardiovascular OR metabolic) AND (blood stasis OR blood stagnation OR blood coagulation OR resolving blood OR activating blood) AND (herbal medicine OR herb). Searches will be conducted in Korea, English, Japanese, and Chinese.

### 2.3 Study selection

#### 2.3.1 Types of studies

We will include all randomized controlled trials (RCTs) or quasi-RCTs evaluating the effectiveness of herbal medicine. Case studies, qualitative studies, uncontrolled trials, and reviews will be excluded, as will trials that fail to provide detailed results.

#### 2.3.2 Types of participants

Participants of both sexes and of any age with clinically diagnosed metabolic disease with blood stasis will be included. Hyperlipidemia, hypertension, diabetes mellitus, metabolic syndrome, and heart diseases such as angina pectoris will be regarded as metabolic diseases. There will be no restriction to diagnostic criteria of blood stasis.

#### 2.3.3 Types of intervention

Any type of herbal medicines, including single herbal extract will be included, with no limitations on number, administration method, dosage, or duration of treatment. Comparators will include no treatment, placebo, and conventional medicines. We will also include trials using other types of herbal medicine for comparison.

#### 2.3.4 Types of outcome measurements

Primary outcomes:

(1) Blood-stasis syndrome score
(2) Total cholesterol (TC), triglyceride (TG), high density lipoprotein cholesterol (HDL-C), low density lipoprotein cholesterol (LDL-C)

Secondary outcomes:

(1) Blood pressure (SBP: systolic blood pressure, DBP: diastolic blood pressure), fasting blood sugar (FBS)
(2) An electrocardiogram (ECG), prevalence rate of heart disease
(3) Response rate

### 2.4 Data extraction and assessment of risk of bias

#### 2.4.1 Data extraction

Two authors (Soobin Jang and Mi Mi Ko) will then perform the data extraction and quality assessment using a predefined data extraction form. Any disagreements arising during the selection and extraction procedures between the authors will be resolved by discussion, or, if agreement cannot be achieved, an arbiter (Jeeyoun Jung) will be consulted and will make the final decision.

#### 2.4.2 Assessment of risk of bias

The risk of bias of the included studies will be assessed using the Cochrane Collaboration's risk of bias assessment tool, version 5.1.0, which considers random sequence generation, allocation concealment, the blinding of the participants and personnel, the blinding of the outcome assessment, incomplete outcome data, selective reporting and other sources of bias. The results of the assessments will be presented as ‘L’, ‘U’, and ‘H’, with ‘L’ indicating a low risk of bias; ‘U’, an uncertain risk of bias; and ‘H’, a high risk of bias.

### 2.5 Data analysis

Quantitative analyses will be performed according to the kind of herbal medicine consisting of same prescription. All statistical analyses will be conducted using the Cochrane Collaboration's software programme Review Manager (RevMan) version 5.3. (Copenhagen, The Nordic Cochrane Centre, the Cochrane Collaboration, 2012) for Windows. Dichotomous data will be reported as risk ratio with 95% confidence intervals (CIs), and continuous data will be reported as mean difference with 95% CIs. For primary outcomes, if the meta-analysis results are significantly heterogeneous, subgroup analysis will be performed as detailed below. We will contact the corresponding authors of the studies for any missing information, and to verify the data whenever possible. If appropriate, we will pool data across studies to conduct a meta-analysis using fixed or random effects models as appropriate, and will also use the GRADEpro software from Cochrane Systematic Reviews to create a summary of findings table.

#### 2.5.1 Assessment of heterogeneity

We will use the random effect model for meta-analysis. If a meta-analysis is possible, we will use the I2 statistic to quantify inconsistencies across the included studies. Heterogeneity was analysed by the Cochrane Q and I^2^ test. I^2^ values were interpreted following the Cochrane Handbook: 0% to 40% may represent a low level of heterogeneity; 30% to 60% may represent moderate heterogeneity; 50%to 90% may represent substantial heterogeneity; and 75% to 100% may represent considerable heterogeneity.

#### 2.5.2 Assessment of reporting biases

If more than 10 studies are available, we will assess funnel plot asymmetry for publication bias and small study effects using Egger method. As funnel plot asymmetry does not necessarily indicate publication bias, we will attempt to distinguish possible reasons for the asymmetry, including poor methodological quality and true heterogeneity of studies.

### 2.6 Ethics and dissemination

Ethical approval is not required, given that this protocol is for a systematic review. The findings of this review will be disseminated widely through peer-reviewed publications and conference presentations.

## 3 Discussion

In summary, the findings of this study will provide a summary of the current state of evidence regarding the effectiveness of types of herbal medicine in managing metabolic disease with blood stasis. In addition, this review will be expected to provide a base for clinical trials to confirm the efficacy of herbal medicine for treatments of metabolic disease with blood stasis.

## Author contributions

**Conceptualization:** Soobin Jang.

**Data curation:** Mi Mi Ko, Soobin Jang.

**Formal analysis:** Mi Mi Ko, Soobin Jang.

**Funding acquisition:** Jeeyoun Jung.

**Investigation:** Jeeyoun Jung.

**Methodology:** Mi Mi Ko, Soobin Jang.

**Supervision:** Jeeyoun Jung.

**Validation:** Jeeyoun Jung.

**Writing - original draft:** Mi Mi Ko, Soobin Jang.

**Writing - review & editing:** Mi Mi Ko, Soobin Jang, Jeeyoun Jung.
